# Relationships between circulating branched chain amino acid concentrations and risk of adverse cardiovascular events in patients with STEMI treated with PCI

**DOI:** 10.1038/s41598-018-34245-6

**Published:** 2018-10-25

**Authors:** Xiaoyu Du, Hongzhao You, Yulin Li, Yuan Wang, Peng Hui, Bokang Qiao, Jie Lu, Weihua Zhang, Shanshan Zhou, Yang Zheng, Jie Du

**Affiliations:** 1 First Hospital of Jilin University, Changchun, Jilin, 130021 China; 2 Beijing Anzhen Hospital, Capital Medical University, Beijing, 100029 China; 3 Key Laboratory of Remodelling-Related Cardiovascular Diseases, Ministry of Education, Beijing, 100029 China

## Abstract

The incidence of in-hospital cardiovascular adverse events (AEs) in patients with ST-segment elevation myocardial infarction (STEMI) following primary percutaneous coronary intervention (PCI) is relatively high. Identification of metabolic markers could improve our understanding of the underlying pathological changes in these patients. We aimed to identify associations between concentrations of plasma metabolites on admission and development of in-hospital AEs in post-PCI patients with STEMI. We used targeted mass spectrometry to measure plasma concentrations of 26 amino acid metabolites on admission in 96 patients with STEMI who subsequently developed post-PCI AEs and in 96 age- and sex-matched patients without post-PCI cardiovascular AEs. Principal component analysis (PCA) revealed that PCA-derived factors, including branched chain amino acids (BCAAs), were associated with increased risks of all three pre-specified outcomes: cardiovascular mortality/acute heart failure (AHF), cardiovascular mortality, and AHF. Addition of BCAA to the Global Registry of Acute Coronary Events risk score increased the concordance C statistic from 0.702 to 0.814 (p < 0.001), and had a net reclassification index of 0.729 (95% confidence interval, 0.466–0.992, p < 0.001). These findings demonstrate that high circulating BCAA concentrations on admission are associated with subsequent in-hospital AEs after revascularization in patients with STEMI.

## Introduction

Primary percutaneous coronary intervention (PCI) is currently the recommended treatment for patients presenting with ST-segment elevation myocardial infarction (STEMI)^1^, salvaged viable myocardium, limited myocardial infarction (MI) size, and preserved ventricular systolic function. However, these patients may develop in-hospital cardiovascular adverse events (AEs) such as cardiac rupture, malignant arrhythmias, and onset of heart failure (HF) after PCI as a result of myocardial ischemia and ischemia–reperfusion injury^2^. The 30-day mortality rate and incidence of in-hospital acute heart failure (AHF) among patients with STEMI who undergo primary PCI are reportedly 7.9% and 28%, respectively^3,4^. An early and efficient interventional strategy could reduce the incidence of cardiovascular AEs and improve these patients’ short- and long-term prognoses^5^.

Current optimal secondary prevention therapies for patients with STEMI who have undergone PCI primarily involve drugs such as antiplatelet agents, statins, β-blockers, angiotensin-converting enzyme inhibitors, and aldosterone antagonists^6^. However, the limited effectiveness of these strategies in preventing and treating post-MI cardiovascular AEs suggests that interruption of the pathways responsible for the AEs are needed to improve outcomes. Maladaptive molecular processes may be a significant cause of such AEs; thus, a metabolomics approach may help to address these problems.

Following primary PCI, there are metabolic changes in the hearts of patients with STEMI and emerging evidence suggests that metabolic remodelling in the heart is the key to development and progression of heart failure^7,8^. Amino acids are important metabolites of nutrients that are vital to survival and growth of cells and serve as substrates for intracellular biosynthesis and signalling molecules^9^. Previous studies have shown that ischemia and ischemia–reperfusion injury lead to disordered amino acid catabolism, especially of the branched-chain amino acids (BCAAs) leucine, isoleucine, valine, and taurine^10–12^. Defective amino acid catabolism sensitizes the heart to ischemia–reperfusion injury, leading to adverse myocardial remodelling and post-MI HF^13^.

In this study, we aimed to assess abnormalities in circulating amino acid metabolites in admission blood samples of patients with STEMI who went on to undergo PCI and who developed in-hospital cardiovascular AEs and compare them with those in age- and sex-matched controls who did not develop AEs.

## Results

### Baseline characteristics of patients with STEMI and post-PCI AEs

Of the 96 patients with STEMI who developed the composite primary outcome of cardiovascular death or AHF during hospitalization, 20 had cardiovascular deaths and 76 AHF. The baseline clinical characteristics of patients who did and did not develop cardiovascular AEs are listed in Table 1. The patients were similarly distributed with regard to sex, age, body mass index (BMI), blood pressure, heart rate, Gensini score, interval between symptom onset and reperfusion^14^, history of hypertension, diabetes mellitus and smoking, and objective laboratory measures, including blood lipids, glucose, and creatine concentrations. Troponin I (TnI) and N-terminal B-type natriuretic peptide (NT-pro BNP) concentrations were significantly higher in the AE than the No-AE group (7.7 ± 19 µg/mL vs. 8.8 ± 6.9 µg/mL, p = 0.001 and 298.5 ± 119.4 ng/L vs. 214.3 ± 80.8 ng/L, p < 0.001, respectively). Furthermore, Global Registry of Acute Coronary Events (GRACE) risk scores were significantly higher in the AE than the No-AE group (131.6 ± 16.9 vs. 145.0 ± 21.8, p < 0.001).

**Table 1:** Baseline clinical characteristics of study patients.

Characteristic	No events (n = 96)	Events (n = 96)	P-value
Male (%)	69 (71.9)	69 (71.9)	1.000
Age, y	59.9 ± 9.8	60.6 ± 11.0	0.620
BMI, kg/m^2^	25.4 ± 2.0	25.8 ± 1.8	0.229
**Blood pressure, mmHg**			
Systolic	132.4 ± 21.7	129.5 ± 21.0	0.534
Diastolic	78.5 ± 13.5	77.3 ± 12.2	0.556
Heart rate, beats/min	75.6 ± 12.1	78.3 ± 13.2	0.174
GRACE risk score	131.6 ± 16.9	145.0 ± 21.8	<0.001
**Comorbidity**			
Hypertension (%)	36 (34.3)	42 (43.8)	0.463
Diabetes (%)	14 (14.6)	20 (20.8)	0.345
Current Smoker (%)	45 (46.9)	47 (49.0)	0.885
Anterior myocardial infarction (%)	42 (43.8)	55 (57.3)	0.083
Symptom onset to reperfusion time, hours	6.3 ± 3.0	6.9 ± 2.9	0.148
**Coronary Angiography**			
1-vessel disease (%)	29 (30.2)	25 (26.0)	0.630
2-vessel disease (%)	30 (31.3)	28 (29.2)	0.875
3-vessel disease (%)	37 (38.5)	43 (44.8)	0.464
Gensini Score	59.8 ± 31.8	64.6 ± 34.8	0.395
**Laboratory examination**			
TCl, mmol/L	4.7 ± 1.0	4.9 ± 1.3	0.635
LDL, mmol/L	2.9 ± 0.8	3.1 ± 0.9	0.509
HDL, mmol/L	1.1 ± 0.3	1.1 ± 0.3	0.343
Glucose, mmol/L	7.0 ± 2.9	7.8 ± 3.5	0.134
Creatine, mmol/L	72.0 ± 19.8	73.8 ± 21.6	0.655
TnI, ng/ml	7.5 ± 9.3	8.8 ± 6.9	0.001
NT-pro BNP, pg/ml	214.2 ± 79.9.	285.0 ± 116.6	<0.001
**Medication**			
Beta-blocker (%)	79 (82.2)	73 (76.0)	0.374
ACEI/ARB (%)	84 (87.5)	87 (90.6)	0.669
Statin (%)	100 (100.0)	100 (100.0)	1.000
IIb/IIIa antagonists (%)	52 (54.2)	59 (61.5)	0.381
IABP implantation (%)	1 (1.0)	4 (4.2)	0.368

Data are presented as mean ± standard deviation or number (%). *P* values derived from Mann–Whitney *U*-tests or χ^2^ tests for continuous and categorical variables, respectively. ACEI = angiotensin-converting enzyme inhibitor; ARB = angiotensin receptor blocker; BMI = body mass index; GRACE = Global Registry of Acute Coronary Events; HDL = high-density lipoprotein; IABP = intra-aortic balloon pump; LDL = low-density lipoprotein; NT-pro BNP = N-terminal pro-B-type natriuretic peptide; TC = total cholesterol; TnI = troponin I.

### Plasma concentrations of 26 amino acids

Differences in admission plasma concentrations of 26 amino acids between the AE and No-AE groups are shown in Supplementary Table 1. Plasma concentrations of valine (p < 0.001), isoleucine (p < 0.001), leucine (p < 0.001), tyrosine (p < 0.001), phenylalanine (p < 0.001), ornithine (p < 0.001), glutamate (p < 0.001), creatine (p < 0.001), creatinine (p < 0.001), serine (p < 0.001), urea (p = 0.006), kynurenine (p = 0.008), and glycine (p = 0.017) were significantly higher in the AE than No-AE group, whereas those of glutamine (p < 0.001), arginine (p = 0.011), and histidine (p = 0.044) were significantly lower in the AE group. Plasma concentrations of the other tested amino acids were similar between the AE and No-AE groups.

### Association between admission amino acid concentrations and in-hospital clinical outcomes

Six meaningful metabolomics factors were identified by principal component analysis (PCA) (Supplementary Table 2): Factor 1 (ornithine, glycine, serine), Factor 2 (BCAAs leucine, isoleucine, valine), Factor 3 (phenylalanine), Factor 4 (urea, creatinine), Factor 5 (taurine), and Factor 6 (threonine).

The associations between these six factors and clinical outcomes were therefore analysed using univariate and multivariate logistic regression models. After adjusting for clinical covariates (including age, BMI, interval between symptom onset and reperfusion, Gensini score, anterior MI, history of hypertension, diabetes mellitus and smoking), Factors 1, 2, and 3 were found to be associated with an increased risk of the composite primary outcome of cardiovascular death or AHF (Factor 1: odds ratio [OR] = 1.73, 95% confidence interval [CI] = 1.12–2.66, p = 0.013; Factor 2: OR = 3.36, 95% CI = 1.98–5.69, *P* < 0.001; Factor 3: OR = 2.35, 95% CI = 1.52–3.61, p = 0.001) (Table 2). With regard to each of the primary outcomes separately, Factor 2 was found to be associated with greater risks of AHF (OR = 2.07, 95% CI = 1.34–3.19, p = 0.001) and of cardiovascular death (OR = 2.22, 95% CI = 1.23–4.00, p = 0.008). Notably, neither Factor 1 nor Factor 3 was associated with risk of cardiovascular death and Factor 1 was also not associated with risk of AHF.

**Table 2:** Univariate and multivariate analyses of associations between metabolomics factors and cardiovascular outcome.

Factor	Univariable		Multivariable*	
	OR (95% CI)	p-Value	OR (95% CI)	p-Value
**Outcome: Cardiovascular Death/Heart Failure**				
1	1.69 (1.23–2.32)	0.001	1.73 (1.12–2.66)	0.013
2	2.97 (1.97–4.46)	<0.001	3.36 (1.98–5.69)	<0.001
3	2.08 (1.50–2.87)	<0.001	2.35 (1.52–3.61)	<0.001
4	1.40 (1.04–1.87)	0.026	1.48 (0.99–2.22)	0.055
5	0.83 (0.62–1.11)	0.209		
6	1.09 (0.82–1.44)	0.567		
**Outcome: Heart Failure**				
1	1.55 (1.14–2.11)	0.005	1.53 (0.99–2.35)	0.052
2	1.90 (1.36–2.66)	<0.001	2.07 (1.34–3.19)	0.001
3	1.77 (1.29–2.42)	<0.001	1.95 (1.26–3.03)	0.003
4	1.21 (0.91–1.62)	0.190		
5	0.90 (0.68–1.20)	0.467		
6	1.08 (0.81–1.43)	0.618		
**Outcome: Death**				
1	1.22 (0.79–1.90)	0.373		
2	1.80 (1.20–2.69)	0.004	2.22 (1.23–4.00)	0.008
3	1.39 (0.87–2.22)	0.174		
4	1.36 (0.88–2.10)	0.169		
5	0.81 (0.53–1.25)	0.346		
6	1.01 (0.64–1.60)	0.965		

Odds ratios indicate a 1-standard deviation increase in factor. *Adjusted for age, BMI, Gensini score, anterior myocardial infarction, symptom onset to reperfusion time, history of hypertension, history of diabetes, and current smoking. Any factor not listed was not significant at *P* ≤ 0.05. CI = confidence interval; OR = odds ratio.

**Figure 1:**
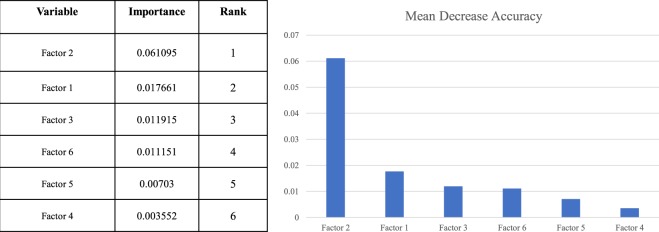
Importance of principal component analysis factors estimated using random survival forest analysis. Variables with the highest importance values were found to be the best predictors of primary clinical outcome.

Random survival forest (RSF) analysis identified the same six metabolomics factors, Factor 2 having the highest value for distinguishing between patients with and without AEs (Fig. 1).

Patients in the highest tertile for Factor 2 were at a 9.45-fold greater risk of developing a primary outcome than those in the lowest tertile (adjusted OR: 9.45; 95% CI = 3.18–28.09, p = 0.001) (Supplementary Fig. 1).

These results suggest that Factor 2 is significantly associated with cardiovascular death and AHF in post-PCI patients with STEMI.

### Associations of traditional biomarkers and PCA factor 2 with in-hospital clinical outcomes

TnI^15^ and NT-pro BNP^16^ are well-established biomarkers for diagnosing MI and predicting primary outcomes. Univariate and multivariate logistic regression models (adjusting for age, BMI, interval between symptom onset and reperfusion time, Gensini score, anterior MI, history of hypertension, history of diabetes, and history of current smoking) identified both NT-pro BNP and Factor 2 as predictors of primary outcomes (adjusted ORs 2.14, 95% CI = 1.652–3.00, *P* < 0.001 and 3.36, 95% CI = 1.98–5.69, p < 0.001, respectively). However, TnI was not identified as a predictor in the current study (OR = 1.29, 95% CI = 0.89–1.29, p = 0.089) (Supplementary Table 3).

### Abilities of NT-pro BNP and Factor 2 to predict in-hospital clinical outcomes

The abilities of NT-pro BNP and Factor 2 to discriminate between patients with and without AEs was determined by calculating the area under (AUC) the receiver-operating characteristic (ROC) curves. According to this analysis, Factor 2 had a slightly higher predictive value than NT-pro BNP (AUC: 0.69 vs. 0.74 for the composite primary outcome) (Supplementary Fig. 2), whereas the combination of NT-pro BNP plus Factor 2 yielded an higher AUC value (AUC 0.82, p < 0.001) for primary outcome (Supplementary Fig. 2).

### Extent of benefit of adding Factor 2 and NT-pro BNP to the GRACE prediction model

GRACE risk score is a recommended in-hospital risk assessment tool^17^. Whether addition of Factor 2 improved risk prediction compared with that provided by GRACE score alone was therefore investigated. Addition of Factor 2 to the GRACE score significantly improved prediction of cardiovascular AEs, increasing the C statistic from 0.702 to 0.814 (p < 0.001); the net reclassification index (NRI) was 0.729 (95% CI, 0.466–0.992, p < 0.001) and the integrated discrimination improvement (IDI) 0.186 (95% CI, 0.131–0.240, p < 0.001). Adding NT-pro BNP only moderately improved prediction by GRACE score, increasing the C statistic to 0.760 (p = 0.069). Importantly, adding a combination of Factor 2 and NT-pro BNP to the GRACE score further improved risk prediction, increasing the C statistic to 0.869 (p < 0.001). The NRI (NRI: 1.000, 95% CI, 0.756–1.244, p < 0.001) and IDI (IDI: 0.306, 95% CI, 0.241–0.371, p < 0.001) were also significantly higher (Table 3).

**Table 3:** C-statistics, NRI, and IDI for incremental predictive values of in-hospital cardiovascular death and acute heart failure following the sequential addition of NT-pro BNP and factor 2 to GRACE score.

Feature	C-Statistics (95% CI)	P-value for improvement	NRI (95% CI)	NRI p-value	IDI (95% CI)	IDI p-value
**Death and HF hospitalization**						
GRACE*	0.702 (0.625–0.778)					
GRACE + NT-pro BNP	0.760 (0.692–0.829)	0.069	0.646 (0.378–0.914)	<0.001	0.095 (0.053–0.136)	<0.001
GRACE + BCAA†	0.814 (0.753–0.876)	<0.001	0.729 (0.466–0.992)	<0.001	0.186 (0.131–0.240)	<0.001
GRACE + NT-pro BNP + BCAA†	0.869 (0.818–0.920)	<0.001	1.000(0.756–1.244)	<0.001	0.306 (0.241–0.371)	<0.001

*GRACE risk score is derived from age, heart rate, systolic blood pressure, creatinine, Killip class, cardiac arrest at admission, presence of ST-segment deviation, and high cardiac enzyme concentrations. *P* values < 0.05 are relative to the GRACE risk model. CI = confidence interval; IDI = integrated discrimination improvement; NRI = net reclassification index.

## Discussion

We examined associations between plasma metabolomics profiles on admission in patients with STEMI and occurrence of in-hospital cardiovascular AEs post-PCI. Metabolite Factor 2, which comprises the BCAAs leucine, isoleucine, and valine, was identified by PCA as independently associated with both cardiovascular death and AHF during hospitalization. Addition of Factor 2 and NT-pro BNP to the GRACE score remarkably improved the predictive value compared with that provided by GRACE score alone.

The association between high admission concentrations of BCAAs and cardiovascular AEs after STEMI may be linked to previously identified correlations between abnormal BCAA catabolism and MI and ischemia–reperfusion injury^13^. BCAAs, including leucine, isoleucine, and valine, are essential amino acids and are acquired from the diet^18^. Although increased dietary protein intake can contribute to high circulating concentrations of BCAAs^19^, recent studies have demonstrated that high plasma BCAA concentrations in individuals with cardiovascular disease are caused by obstructed BCAA catabolism in the myocardium, which results in a build-up of myocardial BCAA that spills over into the circulation^20–22^. The key limiting enzyme of the BCAA catabolic process, branched-chain α-keto acid dehydrogenase (BCKDH), is activated by dephosphorylation with mitochondrial targeted 2C-type serine/threonine protein phosphatase (PP2Cm)^23^. Transcriptomic and metabolomics studies have revealed that expression of *PPM1K* (the gene encoding PP2Cm) is suppressed in cardiomyopathy and that proteins involved in the rate-limiting step of BCAA degradation, including BCKDH E1 α/β and E2 subunits, are downregulated during pathological stress, leading to marked accumulation of branched-chain alpha-keto acids (BCKAs) in stressed heart tissue^24^. Abundant BCAAs directly inhibit mitochondrial respiratory function, leading to superoxide accumulating in the mitochondria of cardiomyocytes^24^. Furthermore, plasma concentrations of BCAAs and BCKAs are significantly increased in *PPM1K* gene knockout mice, whereas in *PPM1K-*null mice cardiac function is compromised at a young age and deteriorates more quickly with mechanical overload^25^. Accumulation of BCAAs has been shown to suppress glucose metabolism and sensitize the heart to ischemic injury^26^. Oral BCAA administration activates mammalian target of rapamycin (mTOR) signalling and exacerbates cardiac dysfunction and remodelling in mice with surgically induced MI^26^. These lines of evidence support the hypothesis that abnormal BCAA catabolism in the ischemic or otherwise pathologically stressed myocardium may cause marked accumulation of BCAAs and BCKAs, which may in turn promote mitochondrial dysfunction, superoxide accumulation, and cardiomyocyte death^9^, resulting in heart failure or death post-MI.

Several other patient-based studies have shown that disturbances of BCAA metabolism are associated with multiple cardiovascular diseases, including coronary artery disease, MI, and HF. Yang *et al*.^27^ have reported that serum concentrations of BCAAs are remarkably higher in patients with coronary artery disease than in healthy individuals, and are independent of diabetes, hypertension, dyslipidaemia, and BMI. Fan *et al*.^28^ demonstrated that plasma leucine concentrations are up-regulated in patients with acute MI compared with patients with unstable angina. A case-control study^22^ showed that plasma leucine and isoleucine concentrations are higher in patients with chronic heart failure than in healthy controls. Several recent studies have investigated the correlation between metabolic disorders of BCAAs and prognosis in patients with cardiovascular disease. Ruiz-Canela *et al*.^29^ reported that high plasma baseline BCAA concentrations are associated with higher cardiovascular disease risk during 1-year follow-up. Furthermore, another recent study^8^ demonstrated that the prognostic value of several metabolites, involving BCAAs, exceeded that of B-type natriuretic peptide in terms of AEs in patients with chronic heart failure.

In the current study, we found that high plasma BCAA concentrations on admission are associated with high risk of in-hospital cardiovascular AEs in patients treated with primary PCI for STEMI. These results suggest that increasing BCAA catabolism to lower plasma concentrations of BCAAs may improve the prognosis of patients with STEMI after PCI. Wang *et al*.^30^ reported finding remarkable impairment of BCAA catabolism in the myocardium in response to infarction in a murine MI model; oral administration of BCAA further increased BCAA concentrations, activated mTOR signalling, and exacerbated cardiac dysfunction and remodelling. Importantly, these researchers showed that inhibition of mTOR by rapamycin or pharmacological inhibition of branched chain keto acid dehydrogenase kinase, a down-regulator of myocardial BCAA catabolism, significantly improves cardiac BCAA catabolism, reduces amounts of myocardial BCAA, and ameliorates post-MI cardiac dysfunction and remodelling. Additional data, including data from experimental studies in larger animals, are needed to clarify whether BCAAs are a potential therapeutic target.

The present study had several limitations. First, this was a small single centre study; our results should therefore be validated in larger cohorts. However, in light of the small samples sizes, we used RSF analysis to validate and increase confidence in our results^31^. Second, the study was also limited by the fact that blood samples were taken in a non-fasting state. Third, our findings are based on blood metabolite profiles, which are not necessarily representative of myocardial metabolism.

In conclusion, we found that high plasma BCAA concentrations on admission are associated with in-hospital cardiovascular AEs in post-PCI patients with STEMI and that addition of plasma BCAA concentrations and NT-pro BNP improves the predictive ability of GRACE risk scores. Of note, BCAAs have been shown to be involved in the pathogenesis of ischemia–reperfusion injury and adverse cardiac remodelling in experimental studies, supporting the hypothesis that disordered BCAA metabolism may represent a novel insight into the pathological mechanisms underlying prognosis after MI. Further studies of BCAA metabolism aimed at improving the prognosis of patients with myocardial infarction and ischemic–reperfusion injury are warranted.

## Materials and Methods

### Study cohort

This is a case-control study of patients with STEMI undergoing primary PCI at the First Hospital of Ji Lin University from January 2014 to January 2017. Details of the study design have been published previously^32^. Briefly, patients >18 years of age referred for primary PCI were enrolled if they met the following criteria: (i) first STEMI; and (ii) onset of chest pain <12 h before presentation. STEMI was defined as continuous chest pain for at least 20 minutes, and new ST-segment elevation ≥0.1 mV in at least two contiguous leads, or new-onset left bundle branch block. Patients with Killip class > I on admission, heart failure, serum creatinine >250 µmol/L, alanine aminotransferase >135 U/L, severe fatty liver or liver cirrhosis, or malignant tumours were excluded, as were patients who had taken amino acid supplements in the preceding 3 months. Aspirin and clopidogrel (both 300 mg) or ticagrelor (180 mg) were administered before catheterization. The postoperative therapeutic strategy was performed in accordance with standard guidelines^1^. The Medical Ethics Committee of the First Hospital, Jilin University approved the study protocol and the study participants provided written informed consent. The primary clinical outcome was the composite variable of cardiovascular death and AHF after PCI during hospitalization. Secondary clinical outcomes included the individual components of the primary outcome separately were also investigated. Cardiovascular death included death from pump failure, arrhythmia, or mechanical complications including ventricular septal rupture and free wall rupture. AHF was defined as presence of dyspnoea, peripheral oedema and pulmonary rales on physical examination, radiological evidence of pulmonary congestion, need for administration of intravenous diuretic agents, or continuous positive airway pressure. A total of 96 patients with STEMI treated with primary PCI who developed in-hospital cardiovascular death or AHF comprised the AE group. We analysed selected metabolites in these 96 AE patients and 96 No-AE patients matched (1:1 ratio) for age group (±3 years) and sex (Supplementary Fig. 3).

### Blood sampling and examination

Blood was drawn from the arterial sheath before coronary angiography and centrifuged at 3000 × g for 10 minutes at room temperature to obtain plasma and then frozen and stored at −80 °C. TnI and NT-pro BNP were measured as described previously^32^.

#### Metabolomics

Amino acid profiling by liquid chromatography–tandem mass spectrometry (LC–MS) was conducted using an Ultimate 3000 UHPLC system coupled to a mass spectrometry system (Q-Enactive MS; Thermo Scientific, Logan, UT, USA),which provided quantitative assessment of concentrations of 26 amino acids. Details of methods can be found in the Data Supplement.

### Traditional biomarker assays

Blood samples were collected from patients with STEMI on admission.

### Calculation of GRACE scores

The GRACE score is widely used to evaluate the risk of death or recurrent MI^33^. In the current study, GRACE risk scores were calculated using age, heart rate, systolic blood pressure, creatinine, Killip class, presence or absence of cardiac arrest on admission, presence or absence of ST-segment elevation, and cardiac enzyme concentrations on admission.

### Statistical methods

Baseline characteristics were compared between the No-AE and AE groups using Mann–Whitney *U*-tests for continuous variables and χ^2^ tests for categorical variables.

PCA was used to reduce the large number of correlated metabolites to a smaller number of uncorrelated factors. Varimax rotation^34^ was used to identify significant factors: only factors with an eigenvalue of ≥1.0 were considered. Individual metabolites with an absolute value for factor load of ≥0.7 are reported as components of a given factor (weighted sum of the standardized metabolites within that factor, weighted on the factor loading for each metabolite). A large cut-off was chosen to avoid false positives. The relationships between PCA-derived factors and clinical outcomes were evaluated using univariate and multivariate logistic regression models, adjusting for traditional STEMI risk factors (age, BMI, symptom onset to reperfusion time, Gensini score, anterior MI, history of hypertension, diabetes, and smoking). ORs and 95% CIs are given.

The PCA results were validated by RSF analysis^35^, this being a powerful machine-learning statistical algorithm for validating results of studies with small sample sizes. PCA factors were used as covariates and events as the outcome. The results were validated by ranking the PCA factors on the basis of importance of the variable as determined by RSF.

Model performance was assessed by calculating the C statistic, NRI, and IDI. The 95% CIs were calculated for each variable. ROC curves were generated and the AUCs calculated to compare the accuracies of traditional biomarkers and the PCA factors. Statistical analyses were performed using commercial software (SPSS software version 25.0, Chicago, IL USA) and R version 3.3.3 (R Foundation for Statistical Computing, Vienna, Austria). A two-tailed *P* value of <0.05 was considered to denote statistical significance.

## Electronic supplementary material

Supplementary Table1,Supplementary Table2
