# Supplementary material for: Relationships between circulating branched chain amino acid concentrations and risk of adverse cardiovascular events in patients with STEMI treated with PCI
Source: Sci Rep. 2018 Oct 25;8:15809. doi: 10.1038/s41598-018-34245-6 (PMC6202350; doi:10.1038/s41598-018-34245-6)
Supplement: Supplementary file 1 — Supplementary Table1,Supplementary Table2 [file 41598_2018_34245_MOESM1_ESM.pdf]

Relationships between circulating branched chain amino acid concentrations and risk of adverse cardiovascular events in patients with STEMI treated with PCI

Xiaoyu Du[1,2]; Hongzhao You[2]; Yulin Li[2,3]; Yuan Wang[2,3]; Peng Hui[1]; Bokang Qiao[2,3]; Jie Lu[2,3]; Weihua Zhang[1]; Shanshan Zhou[1]; Yang Zheng[1*]; Jie Du[2,3*]

Author affiliations:

[1]First Hospital of Jilin University, Changchun, Jilin, 130021, China

[2]Beijing Anzhen Hospital, Capital Medical University, Beijing, 100029, China

[3]Key Laboratory of Remodelling-Related Cardiovascular Diseases, Ministry of Education, Beijing, 100029, China

***Please address all correspondence to:**

Yang Zheng, MD, PhD
Cardiovascular Centre, First Hospital of Jilin University, Jiefang Road, Changchun, 130021, China.
E-mail: zhengyang@jlu.edu.cn
or
Jie Du, MD, PhD
Beijing Anzhen Hospital, Capital Medical University; Key Laboratory of Remodelling-Related Cardiovascular Diseases, Ministry of Education; Beijing Collaborative Innovation Centre for Cardiovascular Disorders; 2 Anzhen Road, , Beijing, 100029, China.
E-mail: jiedu@ccmu.edu.cn

Methods

Metabolomic profiling methodology

We profiled amino acids metabolites using liquid chromatography–tandem mass spectrometry (LC–MS). LC–MS-grade solvents and isotopically-labelled analytical standards were purchased from Sigma-Aldrich (St. Louis, MO, USA) and Cambridge Isotope Laboratories, Inc. (Andover, MA, USA), respectively. Calibration curves were prepared for a subset of the profiled analytes by serial dilution in stock pooled plasma using stable isotope-labelled reference compounds (D5-glutamate, D3-aspartate, [^{13}C4]-asparagine, D3-serine, D5-glutamine, D5-histidine, D5-threonine, D7-alanine, D7-arginine, D7-proline, D8-valine, D3-methionine, D10-isoleucine, D10-leucine, D8-phenylalanine, D8-tryptophan, D9-lysine). Protein was precipitated by the addition of 200 μL aliquots into 800 μL ice-cold acetonitrile containing the appropriate volume of mixed internal standards, followed by vortexing and centrifugation. The metabolite profiles of the plasma samples were analysed by LC–MS using an Ultimate 3000 UHPLC system coupled to a mass spectrometry system (Q-Enactive MS®, Thermo Scientific, Logan, UT, USA). Chromatographic separation was conducted using a Waters HESS T3 column (1.8 mm, 100 mm × 2.1 mm). Both the mobile phase A (water) and phase B (methanol) contained 0.1% formic acid and 5 mM ammonium acetate, with the addition of 0.015% heptafluorobutyric acid to mobile phase A. The linear gradients were: 2% B at 0.5 min, 20% B at 7 min, 50% B at 10 min, 98% B at 10.1 min, 98% B at 14 min, 2%B at 14.1, and 2% B at 17 min. The flow rate was 0.3 ml/min, and the column temperature was set at 40°C. The Q-Exactive MS operation was carried out in positive-ion mode. The resolution of the full scan was set at 35,000, and the scan range was 70–300 m/z. The detailed MS parameters for the amino acid analysis are shown in Supplemental Table 4.

Supplementary Figure 1

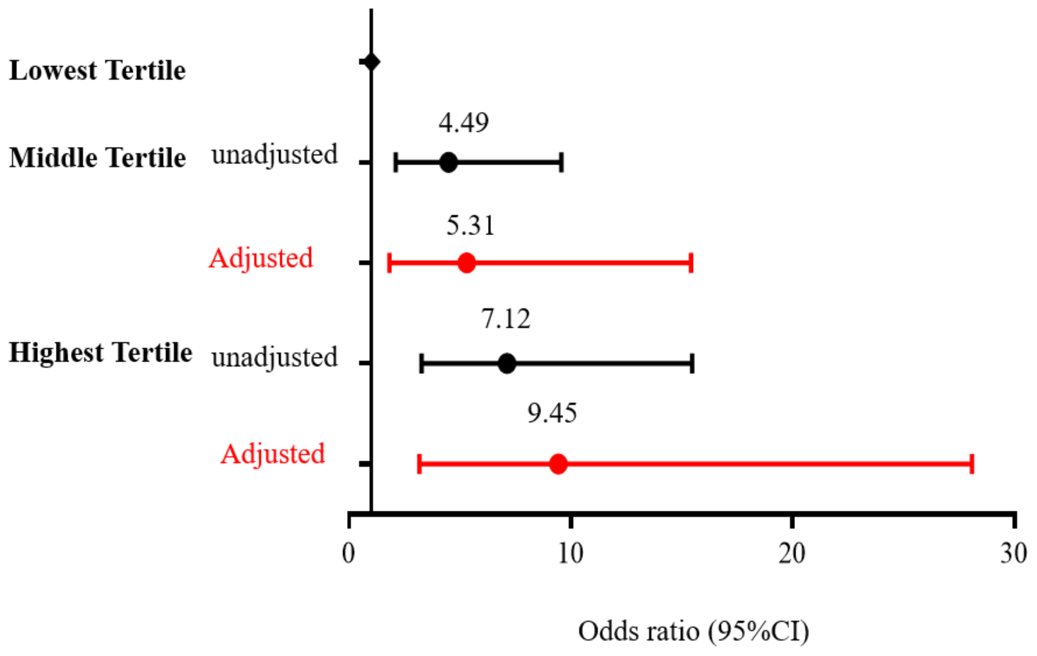

Supplementary Figure 2

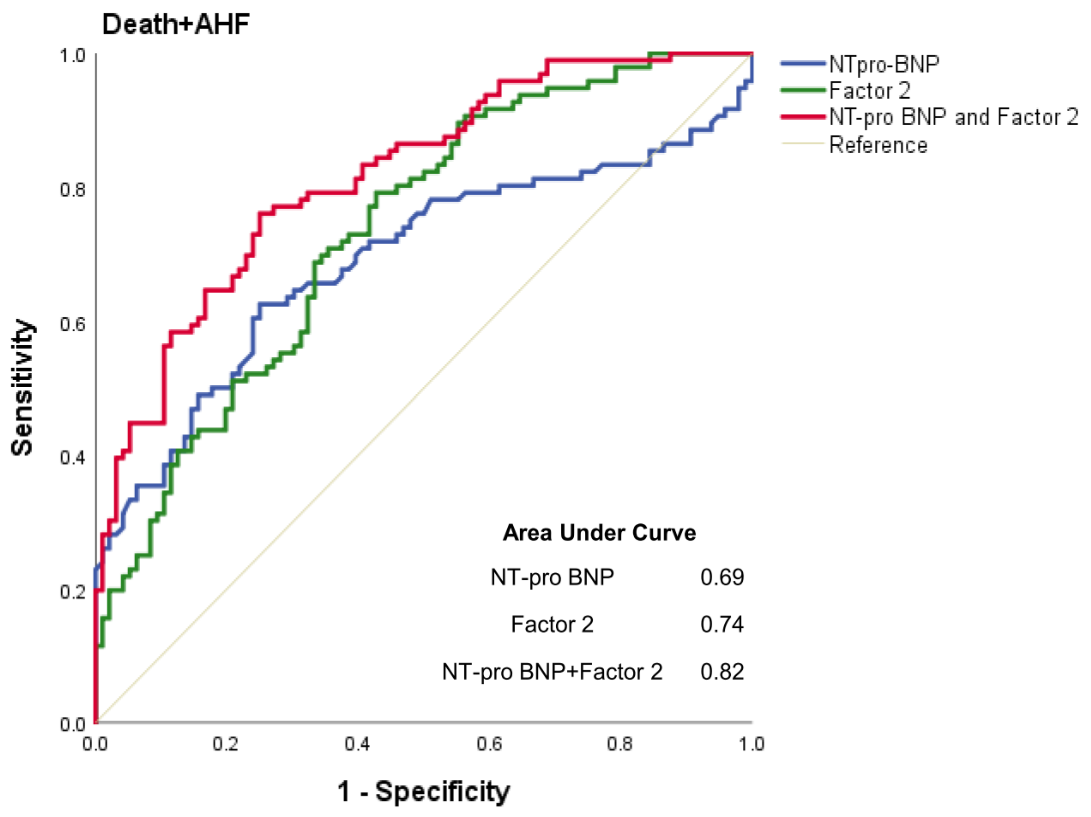

Supplementary Figure 3

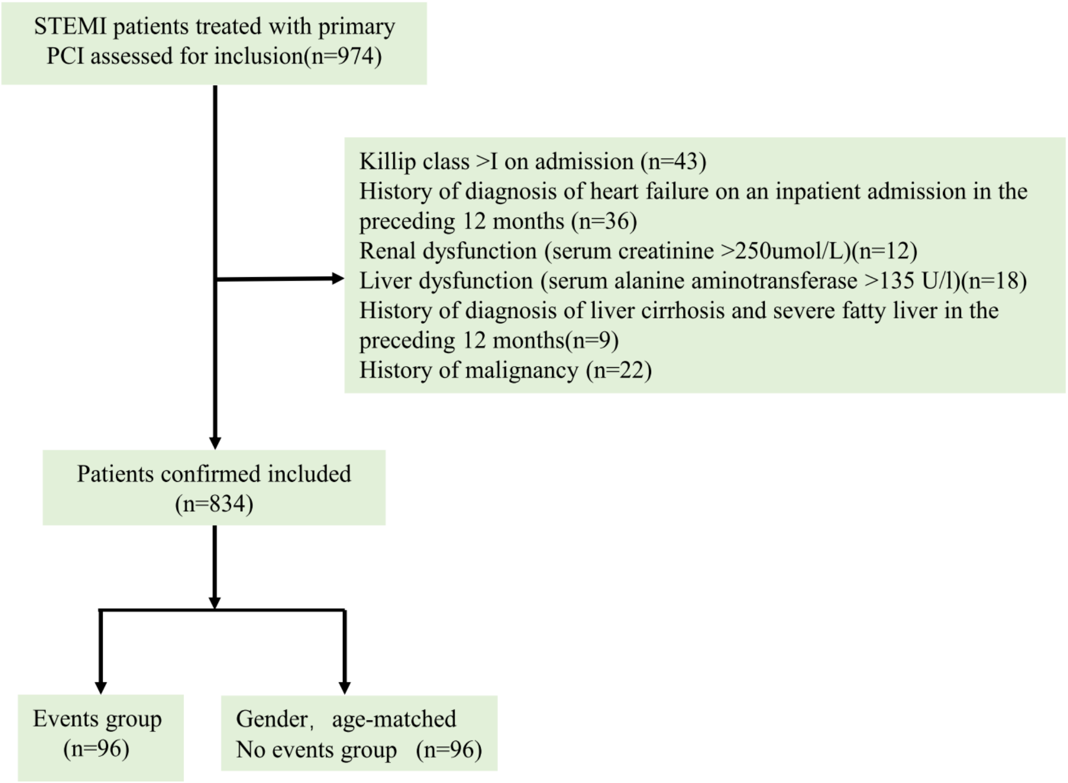

Supplementary Table 1

Metabolites	No events (μmol/L)	Events (μmol/L)	P-value
Valine	212.807±59.631	284.164±71.943	<0.001
Isoleucine	84.181±28.100	111.301±33.031	<0.001
Leucine	145.374±46.854	188.172±51.866	<0.001
Tyrosine	18.177±4.553	22.485±5.061	<0.001
Phenylalanine	58.3521±15.334	70.771±14.412	<0.001
Ornithine	11.647±5.951	17.711±9.500	<0.001
Glutamate	27.563±11.887	39.310±17.231	<0.001
Creatine	55.370±27.531	77.579±39.888	<0.001
Creatinine	338.207±107.522	400.128±101.877	<0.001
Glutamine	270.630±88.380	226.774±66.666	<0.001
Urea	9.172±4.108	11.370±5.377	0.006
Kynurenine	1.233±0.368	1.416±0.522	0.008
Arginine	19.650±5.668	17.822±9.184	0.011
Glycine	123.592±28.014	137.298±40.328	0.017
Histidine	69.239±16.203	64.415±17.539	0.044
Asparagine	34.760±10.163	38.221±13.233	0.108
Alanine	228.467±50.815	242.402±73.034	0.199
Tryptophan	48.966±12.043	52.478±16.403	0.232
Taurine	83.968±327.010	100.572±60.267	0.237
Lysine	38.929±13.331	41.640±16.982	0.344
Citrulline	12.725±4.340	12.357±5.291	0.443
Serine	48.607±10.972	47.776±15.264	0.551
4-Hydroxyproline	1.655±0.615	1.725±0.810	0.581
Threonine	57.823±19.908	65.947±44.372	0.625
Methionine	16.646±4.885	16.407±5.330	0.883
Proline	69.700±20.260	70.247±26.374	0.997

Supplementary Table 2

Factor	Components	Eigenvalue	Variance
1	Orn, Gly, Ser	10.16	0.39
2	Leu, Iso, Val	2.59	0.10
3	Phe	2.09	0.08
4	Ure, Cre	1.65	0.06
5	Tau	1.21	0.05
6	Thr	1.03	0.04

Supplementary Table 3

	Univariable		Multivariable*	
	OR (95%CI)	p-Value	OR (95%CI)	p-Value
TnI	1.29(0.96-1.74)	0.089		
NT-pro BNP	2.08(1.51-2.88)	<0.001	2.14(1.52-3.00)	<0.001
Factor 2	2.97(1.97-4.46)	<0.001	3.36(1.98-5.69)	<0.001

Supplementary Table 4

No.	Metabolite	Identification *m/z*	Retention Time (min)
1	Ornithine	133.09699	1.22
2	Lysine	147.11241	1.28
3	Histidine	156.07646	1.30
4	Glycine	76.039179	1.30
5	Serine	106.04967	1.30
6	Asparagine	133.06042	1.32
7	Taurine	126.02171	1.33
8	Glutamine	147.07611	1.38
9	Alanine	90.05480	1.39
10	Arginine	175.11853	1.39
11	4-Hydroxyproline	132.06532	1.40
12	Threonine	120.06533	1.40
13	Glutamate	148.06009	1.43
14	Urea	61.03974	1.44
15	Citrulline	176.10257	1.48
16	Creatinine	114.06599	1.59
17	Proline	116.07040	1.66
18	Creatine	132.07648	1.68
19	Valine	118.08601	2.52
20	Methionine	150.05815	3.10
21	Tyrosine	182.08086	4.81
22	Isoleucine	132.10168	4.89
23	Leucine	132.10168	4.98
24	Kynurenine	209.09195	5.39
25	Phenylalanine	166.08591	5.42
26	Tryptophan	205.09675	6.01

Figure and Table legends

Supplementary Table 1 Statistical analysis of 26 targeted amino acids between AE group and No AE group. Data are presented as mean ± SD. P values from Mann-Whitney *U*-test.

Supplementary Table 2 Principal Component Analysis. The table lists the 6 metabolomic factors identified by Principal Component Analysis and the associated individual components, eigenvalue and variance. Orn Ornithine; Gly, Glycine; Ser, serine; Leu leucine; Ile, isoleucine; Val, valine; Phe, Phenylalanine; Ure, urea; Cre, creatine; Tau, taurine; Thr, threonine.

Supplementary Table 3 Logistic regression analysis for traditional biomarkers and Factor 2 for in-hospital cardiovascular death and acute heart failure. Odds ratio indicates 1-SD increase. *Adjusted for age, BMI, Gensini score, anterior MI, symptom onset to reperfusion time, history of hypertension, history of diabetes and current smoking.

Supplementary Table 4 LC-MS parameters of amino acids.

Supplementary Figure 1 The association of Factor 2 with cardiovascular AEs. Forrest plot indicating the risk of in-hospital main cardiovascular adverse events (AEs) according to the tertiles of Factor 2 per 1 standard deviation increase, multivariable logistic regression model for odds ratio, after correcting for age, BMI, symptom onset to reperfusion time, Gensini score, anterior MI, history of diabetes, history of hypertension, current smoking. The 5%–95% confidence interval is indicated by line length.

Supplementary Figure 2 Prognostic Values of NT-pro BNP and Factor 2. The ROC curve analysis for comparing the in-hospital cardiovascular AEs prognostic values of

NT-pro BNP, Factor 2 and the combination of both.

Supplementary Figure 3 Workflow of the selection of patients for the study. STEMI, ST-segment elevation myocardial infarction; Events, cardiovascular AEs including in-hospital cardiovascular death and acute heart failure.
